# Increased Brain Iron Detection by Voxel-Based Quantitative Susceptibility Mapping in Type 2 Diabetes Mellitus Patients With an Executive Function Decline

**DOI:** 10.3389/fnins.2020.606182

**Published:** 2021-01-15

**Authors:** Jing Li, Qihao Zhang, Nan Zhang, Lingfei Guo

**Affiliations:** ^1^Department of Radiology, Beijing Friendship Hospital, Capital Medical University, Beijing, China; ^2^Department of Radiology, Weill Cornell Medical College, New York, NY, United States; ^3^Shandong Medical Imaging Research Institute, Cheeloo College of Medicine, Shandong University, Jinan, China

**Keywords:** type 2 diabetes mellitus, magnetic resonance imaging, quantitative susceptibility mapping, iron deposition, executive function

## Abstract

**Purpose:**

Brain iron accumulation has been suggested as a pathomechanism in patients with type 2 diabetes mellitus (T2DM) with cognitive impairment. This research aims to examine the total-brain pattern of iron accumulation in relation to executive function decline in patients with T2DM by voxel-based quantitative susceptibility mapping (QSM) analysis.

**Materials and Methods:**

A total of 32 patients with T2DM and 34 age- and sex-matched healthy controls (HCs) were enrolled in this study. All participants underwent brain magnetic resonance examination, and 48 individuals underwent cognitive function assessments. Imaging data were collected with three-dimensional fast low-angle shot sequences to achieve magnitude as well as phase images. Using voxel-based QSM analysis, we compared the voxel-wise susceptibility values of the whole brain among groups and explored whether the susceptibility values had correlations with cognitive data.

**Results:**

Among the 66 participants, cognitive function was estimated in 23 patients with T2DM (11 males and 12 females; average age, 64.65 ± 8.44 years) and 25 HCs (13 males and 12 females; average age, 61.20 ± 7.62 years). T2DM patients exhibited significantly (*t* = 4.288, *P* < 0.001) lower Montreal Cognitive Assessment (MoCA) scores [T2DM, 27 (27, 28); HCs, 29 (28, 29); normal standard ≥ 26)] and higher Trail-making Test (TMT)-A/TMT-B scores [71 (51, 100)/185 (149, 260)] than HCs [53 (36.5, 63.5)/150 (103, 172.5)] (*Z* = 2.612, *P* = 0.009; *Z* = 2.797, *P* = 0.005). Subjects with T2DM showed significantly higher susceptibility values than HCs in the caudate/putamen/pallidum, frontal inferior triangular gyrus, and precentral gyrus on the right hemisphere. In contrast (HC > T2DM), no region showed a significant difference in susceptibility values between the groups. The correlation analysis between susceptibility values and cognitive function scores was tested by voxel-based susceptibility value with sex and age as covariates. After multiple comparison correction, in T2DM patients, the left thalamus showed a significant relationship with TMT-A (*R*^2^ = 0.53, *P* = 0.001). The right thalamus and left thalamus showed a significant relationship with TMT-B (*R*^2^ = 0.35, *P* = 0.019; and *R*^2^ = 0.38, *P* = 0.017, respectively). In HCs, the cluster of right precentral/middle frontal gyrus/inferior frontal gyrus/inferior triangular gyrus showed a significant relationship with TMT-B (*R*^2^ = 0.59, *P* = 0.010). No relationship was found between the susceptibility values with MoCA in the brain region in both two groups.

**Conclusion:**

Patients with T2DM presented declined cognitive assessments and elevated iron deposition in the striatum and frontal lobe, suggesting that executive function decline in T2DM might be associated with the cerebral iron burden and that changes in susceptibility values may represent a latent quantitative imaging marker for early assessment of cognitive decline in patients with T2DM.

## Background

In type 2 diabetes mellitus (T2DM), peripheral insulin resistance together with compensatory insulin hypersecretion from pancreatic islets likely results in some complications, such as neuropathy, nephropathy, atherosclerosis, and retinopathy (Forbes and Cooper, 2013). Insulin resistance in the brain will lead to subsequent sequelae, which might result in tau hyperphosphorylation and/or amyloid accretion. Insulin functions through the distribution of iron in neuronal tissue; nonetheless, an insulin-resistant state has a disruptive role in the process, consequently resulting in detrimental iron overload (Medhi and Chakrabarty, 2013). Additionally, insulin resistance leads to high permeability of the blood–brain barrier (BBB), and increased permeability with leakage of material into the vessel wall and perivascular tissue will cause inflammation (Wardlaw et al., 2013; Takechi et al., 2017). The inflammatory status of the brain can influence brain iron metabolism and lead to iron deposition (McCarthy et al., 2018). The accumulation of iron in neurons will induce damage by apoptosis (Ward et al., 2014). Therefore, we speculated that the iron excess caused by T2DM can generate damage within the central nervous system.

T2DM patients suffer from cognitive deficits of memory, executive function (EF), attention, visuospatial capabilities, and other domains (McCrimmon et al., 2012). According to one proposal, an early onset of T2DM and weak glycemic control together with microvascular and macrovascular complications may lead to combined cognitive impairment. T2DM can be applied as an individual independent risk factor for Alzheimer’s disease (AD), vascular dementia (VD), and mild cognitive impairment (MCI) (Moon et al., 2016). Patients with T2DM have more executive dysfunction than individuals without diabetes (Minami et al., 2020), which is related to mild-to-moderate EF decreases (Vincent and Hall, 2015). EF decreases in T2DM patients than in healthy older controls but at a smaller scale in AD patients suffering from impairments in executive processing, and T2DM patients may be at a higher risk of developing AD (Redondo et al., 2016).

Heretofore, to image biomarkers, several magnetic resonance imaging (MRI) techniques have been proposed in T2DM patients with cognitive impairment (Zilliox et al., 2016). Because it is easy to provide precise location information of the brain regions for voxel-based morphometry (VBM) (Chen et al., 2017), VBM has been used widely, and gray matter structural and volume alterations of the brain have been revealed to be related to cognitive impairment (Liu et al., 2017). On the basis of a growing number of studies on MRI, brain iron overload has been observed in various neurodegenerative illnesses and is related to cognitive decline (Ward et al., 2014; Del et al., 2015; Daglas and Adlard, 2018; Chai et al., 2019). Quantitative susceptibility mapping (QSM) represents an updated MRI strategy that facilitates quantification of materials by changing susceptibility and enables non-invasive quantitative analysis of brain iron deposition (de Rochefort et al., 2010; Schweser et al., 2012; Wang et al., 2017). The functions of iron in DNA synthesis, gene expression, neurotransmission, myelination of neurons, and the mitochondrial system are considered crucial components in the brain. Therefore, the brain’s iron balance requires strict regulation. Abnormal iron metabolism is relevant to many brain illnesses or disorders. However, the abnormal mode of brain iron accumulation in patients with T2DM has not been fully elucidated *in vivo*. The relationship between iron accumulation and EF decline in T2DM has not been revealed and publicized. Therefore, this research focuses on assessing the whole-brain pattern of iron accumulation in relation to EF decline in patients with T2DM by voxel-based QSM analysis.

## Materials and Methods

### Participants

This cross-sectional research consisted of 32 T2DM patients (20 males; average age, 61.09 ± 9.99 years; age range 39–75 years) and 34 age- and sex-matched healthy control (HC) volunteers (15 males; average age, 58.50 ± 10.07 years; age range, 35–73 years) who were registered from December 2018 to April 2020. The patients all met the diagnostic criteria of T2DM (the diagnosis was based on the American Diabetes Association criteria). In this research, no special selection of T2DM patients according to metabolic control, the existence of micro- or macrovascular complications, neuropathy, the disease duration or treatment type for hyperglycemia, vascular risk factors, or arterial hypertension was applied. The HC volunteers without T2DM had no history of elevated blood glucose levels, and their blood glucose levels were maintained in the normal range (fasting glucose < 5.5 mmol/L). Patients who had a greater than 1-year history of T2DM and willing to undergo the MRI scan were enrolled. The exclusion criteria included a history of psychiatric or neurological disorders (including cerebrovascular accidents), which may affect cognitive functioning, a history of alcohol or substance abuse, acute complications of T2DM (ketoacidosis and severe hypoglycemia) within the 3 months preceding the examination, and MRI scan contraindications. The research obtained approval from the Institutional Review Board of Shandong Medical Imaging Research Institute Affiliated to Shandong University. All of the participants were given information about the experimental procedures and signed consent forms. In consideration of the likely cognitive impairment of the participants, all of the subjects were invited to perform cognitive function assessments according to their educational levels. Finally, 48 people (23 T2DM patients and 25 HCs) completed the questionnaire.

### Clinical Data Collection

The participants underwent an elaborate interview as well as a clinical examination. Age, education level (the number of years in elementary school, high school, and college), and the duration and medical treatment of diabetes were registered and recorded. Systolic blood pressure (BP) and diastolic BP were measured. Arterial hypertension was defined as an average systolic BP > 140 mm Hg and a diastolic BP > 90 mm Hg or self-reported use of medication to lower BP, and fasting glucose, HbA1c fasting triglycerides, and fasting cholesterol were determined by laboratory testing of venous blood samples. Weight and height were measured and used to calculate body mass index (BMI). After the above medical history collection, blood sample collection, and clinical examinations were finished, the participants were invited to visit the clinic on separate days for cognitive tests and MRI scans. The following items were evaluated on the same day, and the interval was 1–4 days between these two visits. The severity of cerebral small vessel disease (CSVD) was assessed using the Fazekas scale (0–3) oriented with periventricular hyperintensity (PVH) together with deep white matter hyperintensity (DWMH) lesions (Fazekas et al., 1987) and by a combined simple CSVD score (0–3 scale, calculated based on the severity of cerebral microbleeds, lacunes, and WMH) (Amin Al Olama et al., 2020).

### Neuropsychological Tests

Standardized general and detailed neurological examinations were conducted on the participators, 48 of whom (23 T2DM patients and 25 HCs) underwent the cognitive assessment, and the assessment tools included the Montreal Cognitive Assessment (MoCA) and the Chinese version of the Trail-making Test (TMT). The MoCA is a one-page 30-point test administered in 10 min (Nasreddine et al., 2005; Bergeron et al., 2017). A score of 13/14 was used as the optimal cutoff point for illiterate individuals, a score of 19/20 was used for individuals with 1–6 years of education, a score of 24/25 was used for individuals with 7 or more years of education, and scores below these cutoff values indicated cognitive impairment (Lu et al., 2011). The subjects also completed the Chinese version of the TMT, which includes two parts: TMT-A to assess cognitive processing speed and TMT-B to measure executive functioning (Woods et al., 2015; Wei et al., 2018). A practice trial with eight items was administered before the actual test to ensure that the individuals understood the tasks. The time required to complete the tasks (in seconds) was recorded as the test score (higher scores indicated lower cognitive function). The test conductor, who had been trained in a professional manner, did not have knowledge about the grouping assignments.

### Image Acquisition

All of the subjects were imaged on a MAGNETOM Skyra 3.0-T MR scanner (Siemens Healthcare, Erlangen, Germany) using a 32-channel head coil for signal reception. The brain scanning protocol consisted of a 3D T1-weighted (T1W) magnetization-prepared rapid gradient echo (MPRAGE) sequence for anatomic structure [repetition time (TR) = 7.3 ms, echo time (TE) = 2.4 ms, inversion time (TI) = 900 ms, flip angle = 9°, and isotropic voxel size = 1 mm^3^]and a 3D multi-echo gradient echo (ME-GRE) sequence for QSM (*TR* = 50 ms, first *TE* = 6.8 ms, *TE* interval = 4.1 ms, number of echoes = 10, flip angle = 15°, and voxel size = 1 × 1 × 2 mm^3^). Additionally, the required sequences also included T2-weighted (T2W) turbo spin echo (TSE), T2W fluid-attenuated inversion recovery (FLAIR), diffusion-weighted (DW), and susceptibility-weighted (SW) imaging to detect brain abnormalities.

### Quantitative Susceptibility Mapping Preprocessing and Quantitative Analysis

Brain QSM maps were computed from complex ME-GRE image data using morphology-enabled dipole inversion with an automatic uniform cerebrospinal fluid (CSF) zero reference algorithm (MEDI + 0) (Liu et al., 2018). Briefly, a non-linear fitting of the multi-echo data was performed to estimate the total field. The total field was spatially unwrapped using a quality-guided region-growing algorithm (Cusack and Papadakis, 2002). Background field removal using the projection onto dipole fields (PDF) algorithm was then applied to compute the local field, which was then inverted to obtain the final susceptibility map. Structural priors (edges) derived from the magnitude image and a regularization term enforcing a uniform susceptibility distribution of the CSF within the lateral ventricles were used in the numerical inversion to improve QSM quality and to provide CSF as an automatic susceptibility reference. The CSF mask was determined by thresholding the R2^∗^ map computed from the GRE magnitude data and imposing voxel connectivity (Liu et al., 2018).

We first acquired gray matter volume images by segmenting the T1 anatomical image through Statistical Parametric Mapping version 12^1^ and resliced QSM images to the same resolution (1 × 1 × 1 mm^3^) as the gray matter volume images. Next, a study-specific brain template was generated on the Diffeomorphic Anatomical Registration Through Exponentiated Lie Algebra (DARTEL) toolbox (Ashburner, 2007). The gray matter volume and QSM maps were then normalized to the Montreal Neurological Institute (MNI) space and smoothed using a Gaussian kernel with an 8-mm full width with half maximum (Pengas et al., 2009).

### Statistical Analysis

The enrolled subjects were separated into two groups (T2DM and HCs). To compare voxel-based QSM values, we used a two-sample *t*-test with sex and age as covariates. A significance cluster-level *P* = 0.05 was applied with correction for multiple comparisons using the family-wise error (FWE) method, and only clusters with sizes > 100 voxels were included. The correlation analysis between susceptibility values and cognitive function scores was tested by voxel-based susceptibility value with sex and age as covariates. A significance cluster-level *P* = 0.05 was applied with correction for multiple comparisons using the FWE method, and only clusters with sizes > 100 voxels were included. The Statistical Package for the Social Sciences (IBM SPSS Statistics for Macintosh, Version 19.0) was used for the statistical analysis. First, descriptive analyses on 32 T2DM patients and 34 HCs were carried out. The measurement data were expressed as the mean ± standard deviation or the median and interquartile range if the data were not normally distributed. The count data were expressed as n (%). The chi-square test was used to compare count data. To compare the clinical data and cognitive assessment scores of the patients with T2DM and HCs, the independent sample *t*-test or the Mann–Whitney *U*-test was used.

## Results

### Participant Characteristics

**FIGURE 1:**
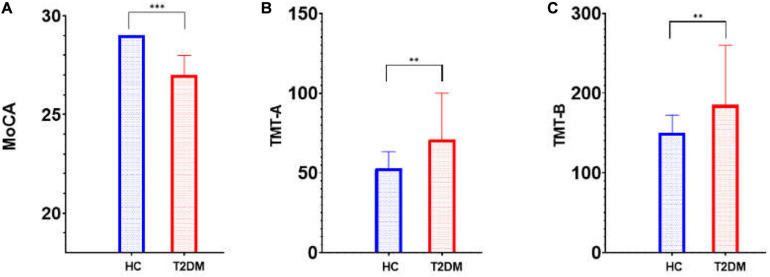
Cognitive score (MoCA and TMT) differences between T2DM patients and HCs. MoCA, Montreal Cognitive Assessment; TMT, Trail-making Test; T2DM, type 2 diabetes mellitus; HC, healthy control. **(A)** MoCA; **(B)** TMT-A; **(C)** TMT-B; ^∗∗^*p* < 0.01; ^∗∗∗^*p* < 0.001.

This research included 32 patients with T2DM (20 males and 12 females with an average age of 61.09 ± 9.99 years) and 34 HCs (15 males and 19 females with an average age of 58.50 ± 10.07 years). These participants showed no significant difference in age or sex (*t* = 1.049, *P* = 0.298; *x*^2^ = 2.236, *P* = 0.133, respectively). Table 1 shows the participants’ clinical features. Of all 66 participants, 23 patients with T2DM (11 males and 12 females; average age, 64.65 ± 8.44 years) and 25 HCs (13 males and 12 females; average age, 61.20 ± 7.62 years) received an assessment of cognitive function. T2DM patients exhibited significantly (*t* = 4.288, *P* < 0.001) lower MoCA scores [T2DM, 27 (27, 28); HCs, 29 (28, 29); normal standard ≥ 26)] and higher TMT-A/TMT-B scores [71 (51, 100)/185 (149, 260)] than HCs [53 (36.5, 63.5)/150. (103, 172.5)] (*Z* = 2.612, *P* = 0.009; *Z* = 2.797, *P* = 0.005). Table 2 and Figure 1 show the scores for each cognitive assessment subindex within these tests and evaluations.

**TABLE 1:** Clinical features of the participants.

	**HC (*n* = 34)**	**T2DM (*n* = 32)**	**Statistical value (*x*^2^ or *t*)**	***P***
Sex (male)	15 (44.12%)	20 (62.50%)	2.236^a^	0.133
Age (years)	58.50 ± 10.07	61.09 ± 9.99	1.049	0.298
Risk factors for cardiovascular disease				
BMI (kg/m^2^)	25.3 ± 4.7	27.5 ± 5.6	1.315^b^	0.104
Fasting serum cholesterol (mmol/L)	5.3 ± 0.9	5.4 ± 1.1	0.398^b^	0.634
Fasting serum triglycerides (mmol/L)	2.2 ± 0.6	2.6 ± 0.8	1. 324^b^	0.117
Hypertension	13 (38.24%)	15 (46.88%)	0.504^b^	0.478^b^
Use of antihypertensive medication	11 (32.35%)	12 (37.50%)	0.192^b^	0.661^b^
History of myocardial infarction	0	0		
CSVD scores	1.1 ± 0.34	1.3 ± 0.45	1.291^c^	0.208^c^
Type 2 diabetes-related factors				
Fasting plasma glucose (mmol/L)	5.2 ± 1.26	9.2 ± 2.44	3.551^b^	<0.001
HbA1c (mmol/ml)	–	61.1 ± 10.3		
HbA1c (%)	–	7.9 ± 1.3		
Diabetes duration (years)	–	11.2 ± 6.5		
Insulin use	–	16 (50.00%)		

*HC, healthy control; T2DM, type 2 diabetes mellitus; BMI, body mass index; CSVD, cerebral small vessel disease. ^*a*^Chi-square test. ^*b*^Independent samples t–test. ^*c*^Mann–Whitney U-test.*

**TABLE 2:** Cognitive functioning assessment of the participants.

**Variables**	**HC (*n* = 25)**	**T2DM (*n* = 23)**	**Statistical value**	***P***
Gender (male)	13 (52.00%)	12 (52.17%)	0.000^a^	0.990
Age, years	61.20 ± 7.62	64.65 ± 8.44	1.847^b^	0.071
Education, years	12.60 ± 2.41	11.34 ± 2.26	1.488^b^	0.144
MoCA	29 (28, 29)	27 (27, 28)	4.288^c^	<0.001
TMT-A	53.00 (36.50, 63.50)	71.00 (51.00, 100.00)	2.612^c^	0.009
TMT-B	150.00 (103.00, 172.50)	185.00 (149.00, 260.00)	2.797^c^	0.005

*HC, healthy control; T2DM, type 2 diabetes mellitus; MoCA, Montreal Cognitive Assessment; TMT, Trail-making Test. ^*a*^Chi-square test. ^*b*^Independent samples t-test. ^*c*^Mann–Whitney U-test.*

### Susceptibility Value Analysis Across Regions of Interest

**FIGURE 2:**
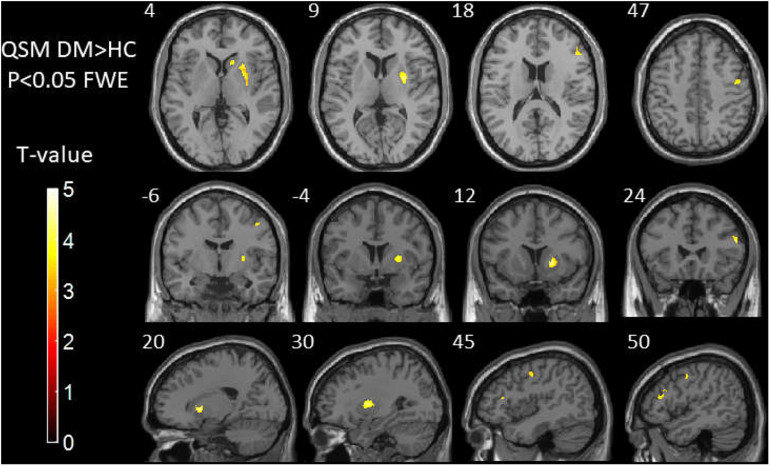
Results of whole-brain voxel-based analyses of susceptibility values between T2DM patients and HCs. The subjects with T2DM showed significantly higher susceptibility values than HCs in the caudate/putamen/pallidum, frontal inferior triangular gyrus, and precentral gyrus on the right side. T2DM, type 2 diabetes mellitus; HC, healthy control.

Table 3 and Figure 2 show the comparison of susceptibility values within whole-brain voxel-based analyses between patients with T2DM and HCs. The subjects with T2DM showed significantly higher susceptibility values than HCs in the caudate/putamen/pallidum, frontal inferior triangular gyrus, and precentral gyrus on the right side. In contrast (HC > T2DM), no region showed a significant difference in susceptibility values between the groups.

**TABLE 3:** Results of whole-brain voxel-based analyses of susceptibility values between T2DM patients and HCs.

**Brain regions**	**Peak MNI (X, Y, Z)**			**Cluster voxels**	***T***	***Z***	***P*^#^**	**Susceptibility value [ppb (× 10^–9^)]**	
								**T2DM**	**HCs**
Right caudate/putamen/pallidum	30	4	9	1,348	−4.52	4.03	0.018	87.84 ± 19.57	63.23 ± 26.02
Right frontal inferior triangular gyrus	53	25	20	591	−4.48	3.99	0.021	15.74 ± 14.44	4.47 ± 9.25
Right precentral gyrus	49	−5	46	221	−3.77	3.37	0.041	8.46 ± 13.00	−3.39 ± 14.19

*T2DM, type 2 diabetes mellitus; HC, healthy control; MNI, Montreal Neurological Institute. ^#^The family-wise error (FWE) method for correction.*

### Correlations of Neuropsychological Test Results With Susceptibility Values

**FIGURE 3:**
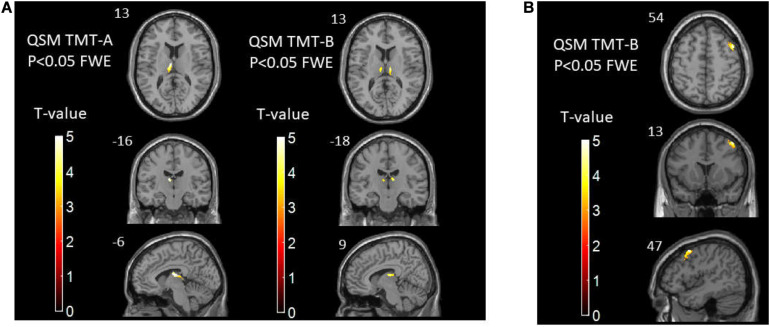
**(A)** The correlations between susceptibility values and TMT-A/TMT-B scores in T2DM patients. **(B)** The correlations between susceptibility values and TMT-B scores in healthy controls. TMT, Trail-making Test; T2DM, type 2 diabetes mellitus.

Correlation analysis between voxel-based susceptibility value and cognitive scores with sex and age as covariates was conducted. A significance cluster-level *P* = 0.05 was applied with correction for multiple comparisons using the FWE method. We found that in T2DM patients, the left thalamus showed a significant relationship with TMT-A (*R*^2^ = 0.53, *P* = 0.001). The right thalamus and left thalamus showed a significant relationship with TMT-B (*R*^2^ = 0.35, *P* = 0.019; and *R*^2^ = 0.38, *P* = 0.017, respectively). In HCs, the cluster of right precentral/middle frontal gyrus/inferior frontal gyrus/inferior triangular gyrus showed a significant relationship with TMT-B (*R*^2^ = 0.59, *P* = 0.010). No relationship was found between the susceptibility values with MoCA in the brain region in both two groups (see Tables 4, 5 and Figure 3).

**TABLE 4:** The correlation using whole-brain voxel-based analyses of susceptibility values in the patients with T2DM.

	**Brain regions**	**Peak MNI (X, Y, Z)**			**Cluster voxels**	***T***	***R*^2^**	***P*^#^**
TMT-A	Left thalamus	−4	−8	19	1,035	4.84	0.53	0.001
TMT-B	Right thalamus	9	−17	14	239	4.28	0.35	0.019
	Left thalamus	−9	−14	14	104	4.49	0.38	0.017

*T2DM, type 2 diabetes mellitus; MNI, Montreal Neurological Institute; TMT, Trail-making Test. ^#^The family-wise error (FWE) method for correction.*

**TABLE 5:** The correlation using whole-brain voxel-based analyses of susceptibility values in the healthy participants.

	**Brain regions**	**Peak MNI (X, Y, Z)**			**Cluster voxels**	***T***	***R*^2^**	***P*^#^**
TMT-B	Right precentral/middle frontal gyrus/inferior frontal gyrus/Inferior triangular gyrus	61	23	27	1,533	4.21	0.59	0.010

*MNI, Montreal Neurological Institute; TMT, Trail-making Test. ^#^The family-wise error (FWE) method for correction.*

## Discussion

**FIGURE 4:**
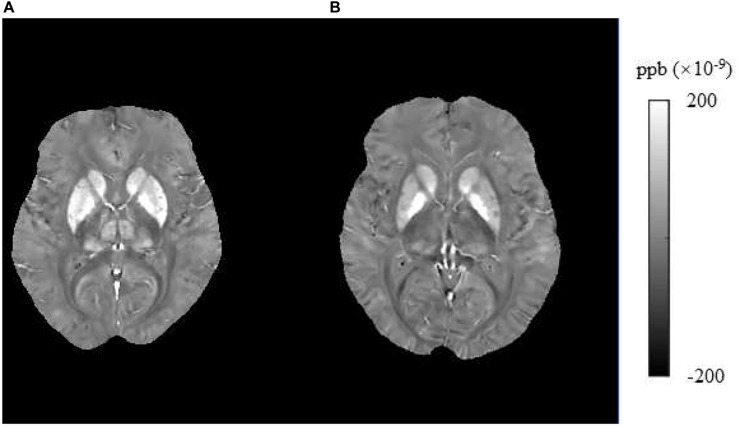
Brain susceptibility value disparities among the patients with T2DM and HCs. **(A)** A patient with T2DM, female, 66 years old, DM history for 15 years. **(B)** HC, female, 67 years old. The bilateral caudate/putamen/pallidum and thalamus presented greater values in T2DM patients than in HCs obviously. T2DM, type 2 diabetes mellitus; HC, healthy control.

**FIGURE 5:**
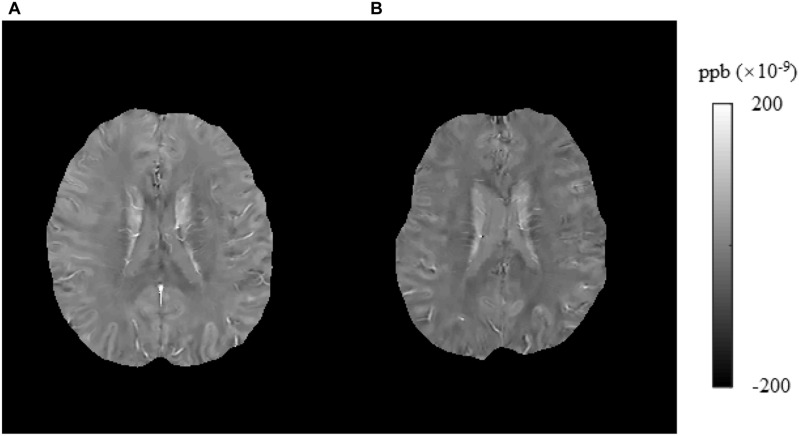
Brain susceptibility value disparities among the patients with T2DM and HCs. **(A)** A patient with T2DM, male, 51 years old, DM history for 10 years. **(B)** HC, female, 57 years old. The right frontal inferior triangular gyrus presented greater values in T2DM patients than in HCs obviously. T2DM, type 2 diabetes mellitus; HC, healthy control.

**FIGURE 6:**
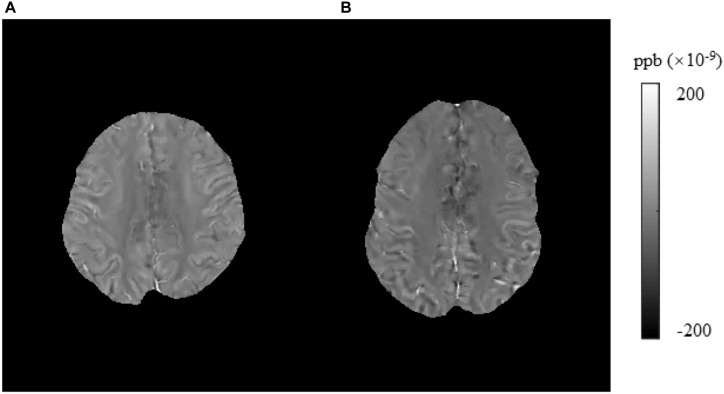
Brain susceptibility value disparities among the patients with T2DM and HCs. **(A)** A patient with T2DM, female, 64 years old, DM history for 15 years. **(B)** HC, male, 61 years old. The right precentral presented greater values in T2DM patients than in HCs obviously. T2DM, type 2 diabetes mellitus; HC, healthy control.

In this study, we found that brain iron in T2DM patients compared with HCs was significantly increased in the striatum containing the caudate/putamen/pallidum and in the frontal lobe (frontal inferior triangular gyrus and precentral gyrus) (Figures 4–6). The increased iron content in multiple brain regions is likely to be related to iron ions, which affect the synthesis of neurotransmitters as well as various metabolic components throughout regions as well as types of neurons (Provost et al., 2015). These brain regions contain major structures that are closely correlated with cognitive, emotional, and motor functions. We also found that T2DM patients exhibited lower MoCA scores and higher TMT-A/TMT-B scores and that the susceptibility values of some brain regions showed significant correlation with TMT-A or TMT-B. These phenomena suggest that the synergy of these changes might have an influence on fronto-striato-thalamic circuits of the brain and then impact EF (Lee et al., 2019; Wu et al., 2020). The results suggest that an increase in iron deposition within the brain might function as a risk factor for the seriousness of brain injury in patients with T2DM.

QSM is a new MRI technique that can measure susceptibility-changing materials quantitatively and accurately. It has the ability to non-invasively analyze the brain iron deposition (de Rochefort et al., 2010; Schweser et al., 2012). QSM is more selective for iron than T2^∗^ relaxometry and can be employed to data from standard acquisition sequences available in most commercial scanners. It is also a computer algorithm that can derive values sensitive to the levels of iron from suitable MRI data (Wang and Liu, 2015; Wang et al., 2017). Iron is an indispensable auxiliary factor involved in oxygen binding and transportation, and energy and material metabolism in the body, which can influence oxygen transportation, cell growth regulation, electron transport, and DNA synthesis. If iron homeostasis is impaired, reactive oxygen species will be excessively produced, resulting in apoptosis (Hubler et al., 2015; Apostolakis and Kypraiou, 2017). Insulin resistance can lead to higher permeability of the BBB. Then, perivascular edema and extravasation of toxic plasma components caused by BBB disruption contribute to localized damage to the brain parenchyma and cognitive dysfunction (Li et al., 2018). T2DM can lead to cerebral small vascular atherosclerosis, which is characterized by smooth muscle cell loss in the tunica media, degeneration of the internal elastic lamina, deposits of fibro-hyaline material and collagens, vessel wall thickening, microatheroma formation, and the obstruction of the lumen (Pantoni, 2010). The occlusion of the arterial lumen leads to lacunar infarction, and the rupture of microatheroma leads to microbleeds. Iron accumulation could occur as an epiphenomenon of demyelination, axonal damage, and/or neurodegeneration (Raz et al., 2015). Histopathologically, microbleeds represent focal accumulations of hemosiderin-containing macrophages (Haller et al., 2018). Therefore, we speculate that cerebral iron overload in patients with T2DM and QSM could reflect this change. Finally, our results confirmed this hypothesis. In this study, we used voxel-based QSM analysis because we consider that a voxel-based analysis is more sensitive than a region of interest (ROI)-based analysis. In our previous study, the differences of the QSM data between T2DM patients and HCs were investigated by ROI-based comparisons using the independent sample *t*-test analysis. Although we found that the brain iron deposits in patients with T2DM had an increasing trend than in healthy elderly individuals in all selected iron-rich gray matter nuclei, after multiple comparison correction, only the susceptibility values of putamen had significant difference (Li et al., 2020).

In this study, using voxel-based QSM analysis, more affected areas were detected, including the striatum containing the caudate/putamen/pallidum and the frontal lobe (frontal inferior triangular gyrus and precentral gyrus). In a previous research, the difference of susceptibility changes caused by iron accumulation was compared among three groups, cognitive normal (CN) elderly, patients with amnestic MCI (aMCI), and patients with early state AD. The differences of the QSM data among the three groups were investigated by voxel-based and ROI-based comparisons. The results of the voxel-based analysis demonstrated more regions with significant difference than the ROI-based analysis (Kim et al., 2017). Therefore, according to the above results, we consider that the voxel-based analysis is more sensitive.

In our study, the affected areas were the striatum containing the caudate/putamen/pallidum and the frontal lobe (frontal inferior triangular gyrus and precentral gyrus). In T2DM patients, the right thalamus and left thalamus showed a significant relationship with TMT scores. In subcortical structures, the thalamus and striatum play key roles in sustaining normal cognitive function. The thalamus functions as an integration center for subcortical and cortical regions; hence, it is a critical component of functions such as awareness and sensory, motor, and cognitive functions. The striatum is one of the main neural structures of the extrapyramidal motor system, which includes the caudate nucleus and lentiform nucleus, the latter of which is divided into the putamen and pallidum (Hedden and Gabrieli, 2010). The striatum plays an indispensable role in various brain functions, including motor control and learning, language, cognitive functioning, and addiction, through functional cortico-striato-thalamocortical neural pathways (Koikkalainen et al., 2007; Fazl and Fleisher, 2018). Therefore, a pathologic state in the striatum may engender a wide range of clinical manifestations such as motor dysfunction and psychiatric disorders (Uono et al., 2017). The frontal inferior triangular gyrus and precentral gyrus are also closely related to the executive control network (Kozlova et al., 2019; Syan et al., 2019). Previous studies have shown that the impaired biophysical completeness of brain macromolecular protein pools and their local microenvironments in the fronto-striato-thalamic circuits in T2DM patients might provide insights into the neurological pathophysiological potential of diabetes-related clinical and cognitive deficits (Yang et al., 2015). According to this information, we thought that the iron overload areas detected in our study could explain the cognitive decline in patients with T2DM.

According to a prior investigation on iron deposition in T2DM patients’ brains using QSM as well as the relevant cognitive impairments, T2DM patients with MCI exhibited significantly increased susceptibility values in the left putamen than T2DM patients without MCI. The susceptibility values of the left putamen showed a close connection to neuropsychological cognitive scores (Yang et al., 2018). Our research also revealed that the susceptibility values of the putamen in T2DM patients were significantly greater than those in healthy elderly individuals. We also found that the MoCA and TMT scores of T2DM patients were markedly lower and higher, respectively, than those of HCs, suggesting that these related cognitive functions of T2DM patients were significantly decreased. Additionally, using voxel-based data, we found in T2DM patients that the left thalamus showed a significant relationship with TMT-A. The right thalamus and left thalamus showed a significant relationship with TMT-B, while in HCs, the cluster of right precentral/middle frontal gyrus/inferior frontal gyrus/inferior triangular gyrus showed a significant relationship with TMT-B. In a large sample research, involving more than 600 older individuals with a mean age of 73 years, the associations between iron deposits and cognitive ability remained consistent significant after controlling for the presence or absence of five health risk conditions that relate to vascular health (hypertension, diabetes, hypercholesterolemia, cardiovascular disease, and occurrence of a previous stroke) (Del et al., 2015). This result and our results strengthen the hypothesis that brains with iron overload have a close relationship with cognitive decline (Fernández-Real and Manco, 2014). Our discoveries suggest that enhanced iron deposition within the brain might be a risk factor for brain injury severity in patients with T2DM. With the use of T1W structural MRI scanning, shape analysis revealed that T2DM is associated with focal atrophy in the bilateral caudate head and dorso-medial part of the thalamus. ROI-based volumetry detected thalamic volume reduction in T2DM when compared with the controls. Furthermore, worse performance of cognitive processing speed correlated with more severe GM atrophy in the bilateral dorso-medial part of the thalamus (Chen et al., 2017). With the use of diffusion kurtosis imaging, a study found that mean kurtosis (MK) decreased in bilateral thalamus and caudate in atlas-based analysis and that MK values correlated with neuropsychological scores in the cingulum (hippocampus) (Xiong et al., 2019). These discoveries and our results clarify that the thalamus is a critical structure for cognitive functions in T2DM patients. In HCs, we found that the cluster of right precentral/frontal middle frontal gyrus/inferior frontal gyrus/inferior triangular gyrus showed a significant relationship with TMT-B. EF is a collection of cognitive processes essential for higher order mental function. Processes involved in EF include working memory, attention, cognitive flexibility, and impulse control. These complex behaviors are largely mediated by prefrontal cortical function (Logue and Gould, 2014). Our result also illustrated that the brain damage in frontal cortex could cause worse EF.

Our study has several limitations. First, there exists background error and white noise in the original susceptibility maps. Therefore, the cluster in the precentral gyrus with susceptibility value close to 0 is subject to the systematic error of QSM reconstruction or white noise. Second, the susceptibility maps normalized to MNI space may suffer from error introduced in VBM pipeline. We found a slightly lower susceptibility values of deep gray matter (DGM) as compared with other studies. Low susceptibility in DGM is also reported in two previous voxel-based QSM analyses (Kim et al., 2017; Uchida et al., 2019). One possible reason is the data smoothing procedure when normalizing to MNI space in the VBM pipeline. This normalization step may introduce other errors. In this study, the individual QSM images are first aligned to individual T1 images and then registered to the study-specific T1 template, which may decrease the contrast in regions such as the basal ganglia and thalamus, where QSM images show high contrast. A study-specific QSM template generated with the T1 template by merging these two modalities may help to solve this problem (Hanspach et al., 2017). Third, this research included an initial cross-sectional investigation of brain iron changes in T2DM patients in a relatively small sample size; on this basis, we should observe iron deposition dynamics by examining longitudinal brain iron levels in patients with T2DM in larger samples at various phases. Prospective research with larger sample sizes is required to confirm the regional susceptibility changes and for in-depth exploration of the latent mechanism to confirm the role of iron deposition in whole-brain pathology.

## Conclusion

In sum, those suffering from with T2DM presented enhanced iron deposition within the striatum and frontal lobe, suggesting that the EF decline in T2DM might be associated with the cerebral iron burden. Using voxel-based data, we identified that the mean susceptibility values of the thalamus, striatum, and frontal lobe had significant correlation with TMT scores, which might reflect a key role of iron deposition during the T2DM process. The changes in susceptibility values throughout these areas likely represent quantitative imaging markers of central nervous system injury among patients with T2DM. Furthermore, QSM might be a beneficial tool for the detection and evaluation of such injuries.

## Data Availability Statement

The raw data supporting the conclusions of this article will be made available by the authors, without undue reservation, to any qualified researcher.

## Ethics Statement

The studies involving human participants were reviewed and approved by the Institutional Review Board of Shandong Medical Imaging Research Institute Affiliated to Shandong University. The patients/participants provided their written informed consent to participate in this study.

## Author Contributions

JL and LG wrote the main manuscript text. QZ prepared imaging data and Figures 1–4. NZ prepared the clinical data. LG revised the main manuscript text. All authors reviewed the manuscript.

## Conflict of Interest

The authors declare that the research was conducted in the absence of any commercial or financial relationships that could be construed as a potential conflict of interest.

## Footnotes

1 http://www.fil.ion.ucl.ac.uk/spm/
