# Supplementary material for: Increased Brain Iron Detection by Voxel-Based Quantitative Susceptibility Mapping in Type 2 Diabetes Mellitus Patients With an Executive Function Decline
Source: Front Neurosci. 2021 Jan 15;14:606182. doi: 10.3389/fnins.2020.606182 (PMC7843466; doi:10.3389/fnins.2020.606182)
Supplement: Supplementary file 1 [file Table_2.DOCX]

**Supplementary Material**

Table1 The susceptibility value diﬀerences [ppb (×10^-9^)] in gray matter nucleus

| DGM structures | HC (n=34) | T2DM (n=32) |
| --- | --- | --- |
| Pallidum | 109.33±36.03 | 121.72±29.54 |
| Putamen | 38.66±19.08 | 52.55±16.86 |
| Caudate nucleus | 25.17±12.46 | 28.03±10.99 |
| Thalamus | -1.95±14.14 | 2.67±1.42 |
| Red nucleus | 179.18±36.59 | 168.55±37.11 |
| Substantia nigra | 179.60±42.89 | 177.43±40.58 |

The mean susceptibility values in Thalamus, Pallidum, Putamen, Caudate nucleus were obtained the susceptibility values of every voxel based on Montreal Neurological Institute (MNI) space. Red nucleus, Substantia nigra were traced directly on the QSM images by a neuroradiologist with 16 years of experience using ITK-SNAP v3.8 software.

Table 2 The susceptibility value in three regions in cluster of “Right Caudate/Putamen/Pallidum”

| DGM structures | DM | HC |
| --- | --- | --- |
| Caudate nucleus | 84.23±21.27 | 64.30±21.68 |
| Putamen | 86.84±19.23 | 62.02±23.65 |
| Pallidum | 118.85±23.43 | 93.19±29.41 |
